# Ultrasound Elastography and Tonometry as Predictive Tools for Capsular Contracture After Breast Implant Surgery: Over a 12-Month Prospective Study

**DOI:** 10.3390/jcm14228084

**Published:** 2025-11-14

**Authors:** Mikołaj Kubasik, Alicja Rzymska, Beata Pięta, Paweł Rzymski

**Affiliations:** 1 Zlotowska Plastic Surgery Clinic, 60-189 Poznan, Poland; 2 The Student Scientific Society (STN), Poznan University of Medical Sciences, 60-355 Poznan, Poland; 3 Department of Practical Midwifery Education, Poznan University of Medical Sciences, 60-355 Poznan, Poland; 4 Department of Mother’s and Child’s Health, Poznan University of Medical Sciences, 60-355 Poznan, Poland

**Keywords:** capsular contracture, shear wave elastography, breast implant, baker score, ultrasound, breast augmentation

## Abstract

**Background/Objectives**: Capsular contracture remains the most common complication after breast augmentation. The Baker score is a classical, yet subjective method to assess the contracture, therefore more objective and reproducible measures are necessary to not only evaluate but also to predict a capsule formation. The aim of the prospective study was to assess the predictive value of sonoelastography, tonometry and physical examination at different time points after the surgery and with association with Baker score, since this early period is considered critical for the initiation of capsular formation. **Methods**: 28 patients (range of age 21.0–40.0) after breast augmentation completed the study protocol. A total of 56 breasts underwent clinical (modified Baker score), tonometric, and elastographic evaluation before surgery, on postoperative days 7 and 14, and after one year. Measurements were taken at the boundaries of the four breast quadrants and included adipose, glandular, muscular and fascial tissues. **Results**: At long-term follow-up, tonometric and elastographic values were lower than on postoperative days 7 and 14. Early measurements in certain quadrants and tissue regions showed some association with Baker scores at one year. All tissues increased in stiffness postoperatively and remained stiffer at the 1-year follow-up. No significant differences in tissue stiffness were observed between days 7 and 14, except for higher values in adipose tissue in the inner quadrant and lower values at the peri-implant region in the outer quadrant. **Conclusions**: Sonoelastography is a precise and objective tool for detecting capsular changes after breast augmentation and may improve early prediction of contracture.

## 1. Introduction

Breast augmentation remains the most common aesthetic surgical procedure worldwide, with an estimated 1.5 million breast implants placed annually. In the United States alone, more than 306,000 augmentations and over 100,000 reconstructions were carried out in 2024, representing a 59% increase compared to the year 2020, according to the American Society of Plastic Surgeons [1,2,3,4,5].

When body identifies the breast implant as a foreign material, an immune response stimulates fibroblasts transforming into myofibroblasts, production of new collagen fibers within the surrounding capsule and proliferation of macrophages and T-CD4+, which leads to the development of a fibrous capsule around the implant [6,7,8,9]. In some cases, exaggerated responses cause capsule contraction (CC), resulting in a hardened and distorted breast shape, with higher Vimentin and α-SMA levels indicating more collagen and thicker capsules [4,7]. The incidence of capsular contracture ranges from 5% to 25%, with an average of around 10% [10]. A 10-year study by Allergan reported a capsular contracture rate of 9.2% following aesthetic augmentation and 14.5% after reconstructive procedures [11]. Most cases of capsular contracture after breast augmentation occur within the first 12 months [12]. Modifications in gel filling, cohesivity, shell surface and thickness influence not only perceptible differences but also long-term complications such as capsular contracture [3,4,7,13,14]. CC is a multifactorial complication, in which biofilm formation from skin- or nipple-associated bacteria plays a central role. Although smooth implants are theoretically less prone to bacterial adhesion, clinical evidence from meta-analyses paradoxically suggests that textured implants may be associated with lower rates of contracture [6,7,12,15,16,17].

The placement of breast implants also influences the risk of capsular contracture. Implants positioned in the subglandular plane show 2-fold higher risk compared to submuscular or subfascial placements [3,13,18,19]. This is likely because the additional tissue layers separating the implant from the mammary gland provide a protective barrier against contracture [6]. Moreover, using the inframammary fold incision during breast augmentation is associated with the lowest risk of capsular contracture (0.59%) compared to other surgical approaches like peri-nipple-areolar incisions (9.5%), as it reduces contact with bacteria colonizing the milk ducts [6,14,16,17].

Breast implants are mainly classified as round or anatomical. Round implants are segments of a sphere and provide enhanced fullness in the upper pole and maintain breast shape regardless of rotation [20], however due to stronger lifting of the muscle and therefore greater tissue irritation and inflammation, they may cause a thicker capsule. They are also more prone to dislocate with lower pole expansion, depending on the elasticity of the surrounding tissue [21]. Anatomical implants, on the other hand, are shaped like a teardrop or half-drop. They offer a very natural appearance, but rotation can distort breast contour, often necessitating surgical correction [5]. The risk of rotation is a commonly cited issue, ranging from 0.9% to 14%, and it is negatively correlated with the time since implantation [5,20]. Recently, autologous fat grafting has become increasingly popular in both reconstructive and aesthetic breast procedures, with 3D imaging, MRI, and ultrasound enabling accurate assessment of graft volume and retention [22].

There are two main methods to evaluate capsular contracture: classical clinical Baker score and modified Baker score, but other techniques like ultrasound elastography seem to be more objective classification [6,8,10,14,16,22,23,24,25,26]. Two main techniques are used in breast elastography: shear-wave elastography (SWE), which quantitatively measures lesion stiffness and strain elastography (SE), a relative method comparing lesion stiffness to surrounding tissues [27,28,29]. Optimized 2D SE techniques achieved 100% sensitivity and allowed reclassification of BI-RADS 4A+ lesions as benign, reducing unnecessary biopsies [28,29,30]. This ability of elastography to detect subtle tissue changes may also be useful for assessing peri-implant capsules and predicting capsular contracture. Magnetic resonance elastography is rarely used because of its high costs and limited accessibility, although it remains the gold standard for evaluating tissue volume and detecting complications such as oil cysts or necrosis after autologous fat transplantation [22].

## 2. Materials and Methods

### 2.1. Study Group

The prospective study included 35 female patients, of whom 28 ultimately completed the entire study protocol. The participants were aged mean 31 ± 5.0 years (median 31.0; range 21.0–40.0) and were recruited from women voluntarily presented to the plastic surgery clinic for cosmetic breast augmentation with implants. This was an interventional study conducted without a control group, as previous data from our study indicated that breast tissue elasticity remains generally stable over time [31]. Participation in the study was voluntary and all patients provided informed consent. The study received a positive opinion from the Bioethics Committee of the Poznan University of Medical Sciences (approval no. 48/16).

### 2.2. Study Protocol

**Figure 1:**
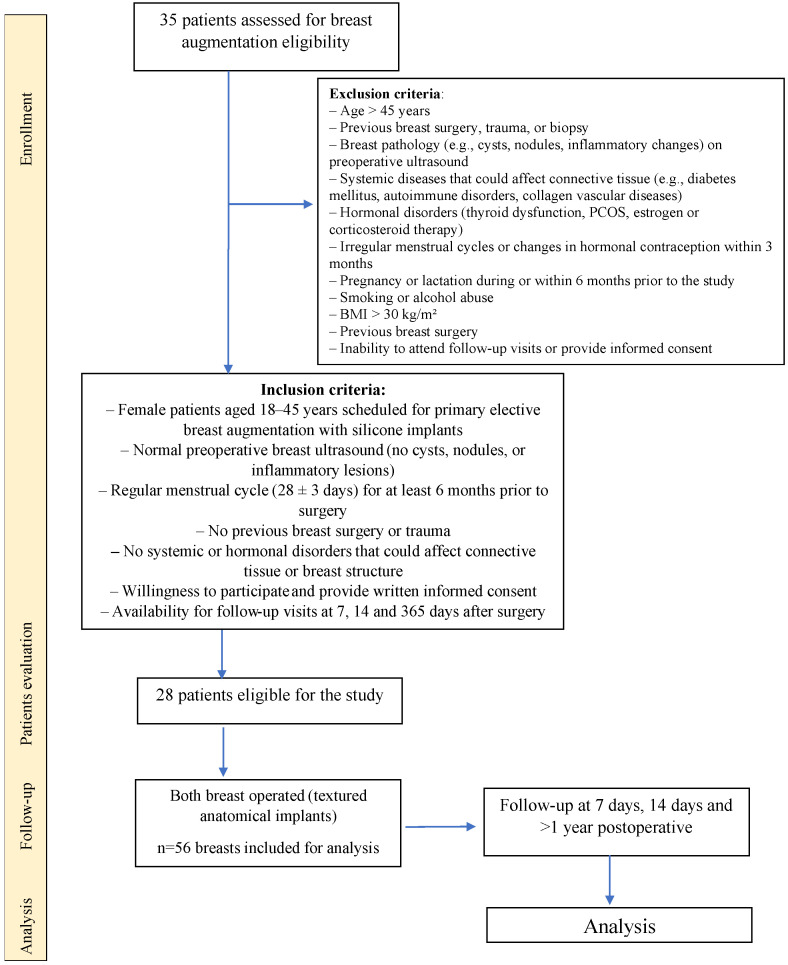
Study protocol flowchart.

Patients underwent preoperative evaluation (first assessment, Visit 1—V1) followed by postoperative follow-up visits. The second visit (V2) took place 7 days after breast augmentation surgery, the third visit (V3) occurred 14 days postoperatively and the final follow-up (V4) was conducted at least 12 to 24 months after the procedure (Figure 1).

The 1- and 2-week postoperative time points were selected with the intention of identifying potential predictive value of the assessed parameters during the early wound healing phase, in relation to long-term outcomes evaluated at Visit 4 (V4). This early period is considered critical for the initiation of capsular formation. The follow-up period ranged from 12 to 24 months, with a mean duration of 14.4 months.

### 2.3. Clinical Assessment

Each visit included a physical examination (palpation) and imaging assessments: breast tissue tonometry, ultrasonography and elastography. The surgical outcome and the presence of capsular contracture were evaluated clinically and graded using a modified Baker classification (grades IA and IB added to address the specific characteristics of reconstructed breasts) [6,8,10,14,16,23,24,25,26]. We treated Baker grade IA as a value of 1.0 and grade IB as 1.5 to reflect the clinical nuance introduced by the subdivision. This approach allowed us to preserve the ordinal nature of the scale while accounting for the subtle differences in capsular contracture severity between IA and IB.

### 2.4. Tonometric Assessment

Breast stiffness was measured using applanation tonometry (UMP, Poznan, Poland) with a standard glass disk (weight: 213 g; diameter: 20.3 cm). Intra-breast pressure (P) was calculated as P = F/A, where P is pressure (N/m^2^), F the applied force (9.8 N/kg) and A the disk’s contact area.

Measurements were performed in the supine position with the disk placed on each breast in its natural position. A brown antiseptic solution (Braunol, B. Braun) was applied prior to obtaining breast imprints, which were subsequently scanned (Brother DCP-395 scanner, Brother, Nagoya, Japan). The imprint area was analyzed in Adobe Photoshop 21.2.2 and expressed as the percentage of pixels relative to a 100 cm^2^ reference standard. Using this method, the total contact area of each breast imprint was automatically determined in all patients. Breast tissue tonometry and palpation performed by a surgeon with 17 years of experience (MK) were independently blinded to the ultrasound and elastography results obtained by the sonographer with 15 years of experience (PR).

### 2.5. Elastographic Assessment

Shear wave elastography (SWE) enables the assessment of tissue stiffness (Young’s modulus), displayed as color-coded images of individual tissue layers: glandular, adipose, fascial, muscular and peri-implant regions. Measurements were taken at the boundaries of the four breast quadrants (LQ—lower quadrants, UQ—upper quadrants, OQ—outer quadrants, IQ—inner quadrants). A 2 mm region of interest (ROI, minimum in the used equipment) was placed at the tissue-implant interface. In the inner and upper quadrants, elasticity measurements included adipose, glandular, muscular and fascial tissues. In the lower and outer quadrants only adipose and glandular tissue elasticity was assessed due to anatomical constraints.

Capsule thickness (minimum and maximum, expressed in mm) was routinely measured using harmonic imaging (HI) during the fourth visit (V4). A capsule thickness exceeding 2 mm was classified as Baker grade III/IV. All elastographic and sonographic assessments were performed using the Aixplorer (SuperSonic Imagine, Aix-en-Provence, France); linear probe, elastographic frequency 100 Hz to 2000 Hz. Previous literature has shown that shear wave elastography generally demonstrates high intra-observer reliability (ICC > 0.9) and moderate inter-observer agreement (ICC~0.7) in breast tissue evaluation [32].

### 2.6. Surgical Technique

The procedure was performed under intravenous anesthesia with the patient in a standard position, after disinfection and draping. Skin incisions were made in the lower areolar region and submuscular pocket was created—dual-plane technique, commonly used implant pocket location [13,18,25]. A 12 Fr drain was placed laterally and the pocket was irrigated with Betadine (povidone–iodine). The same steps were repeated contralaterally. Implants were soaked in an antibiotic solution (Dalacin C 150 mg/mL, Metronidazole Polpharma 0.5%) prior to insertion. Implants were placed and the wounds closed in layers with absorbable sutures. Steri-Strips and dressings were applied and the patient was fitted with a supportive bra and bandage after awakening [13,18,25].

### 2.7. Statistical Analysis

The obtained results were collected in a spreadsheet and analyzed using Statistica v.10 (StatSoft Corp., Fort Lauderdale, FL, USA) and SigmaPlot v.13.0 (Systat, Palo Alto, CA, USA). Due to the non-Gaussian distribution of the data (as assessed by the Shapiro–Wilk test), unequal group sizes, or the use of ordinal-scale variables, statistical analyses were performed using nonparametric methods, as estimated by Shapiro–Wilk test. Differences between two independent groups were evaluated with the Mann–Whitney U test, whereas differences between paired variables were analyzed using the Wilcoxon signed-rank test. Correlations between two variables were examined using Spearman’s rank correlation and Pearson coefficient. Logistic regression analysis was applied in several aspects. The frequency of categorical variables was compared using Pearson’s chi-square test. Results are presented as mean ± SD if not otherwise specified. A *p*-value < 0.05 was considered statistically significant.All graphs were generated using GraphPad Prism v5.0 (GraphPad Software, Boston, MA, USA).

## 3. Results

Final analysis included a total of 28 patients with a mean age of 31 years. All underwent bilateral breast augmentation and each breast was assessed separately (clinically, ultrasonographically, elastographically and tonometrically), resulting in 56 breasts analyzed. Anatomical-type textured implants of models M2, M3, and MX from Allergan (Dublin, Ireland) and Eurosilicone (Apt, France) were used. The mean implant volume was 329.0 ± 71.3. The age of the patients was not significantly associated with the clinical assessment of the capsule according to the Baker scale (Rs = 0.13, *p* > 0.05) and also had no impact on the tonometric parameters measured before and after surgery (V1–V4).

The overall tonometric assessment of breast stiffness in the V1–V4 is presented in Table 1. At long-term follow-up (≥12 months, V4), stiffness values were significantly lower than on V2 and V3 (Wilcoxon test, *p* < 0.05). Breast stiffness was significantly higher at all postoperative time points compared to preoperative tonometric values (Wilcoxon test, *p* < 0.05 for all comparisons), statistically significant and strong correlations were demonstrated (V1:V2, V1:V3, V1:V4 Rs = 0.68, 0.62 and 0.62, respectively, *p* < 0.05). A Paerson correlation and linear regression analysis were conducted to evaluate the relationship between preoperative tonometry values (V1) and postoperative measurements at three time points (V2–4). Pearson correlation coefficients revealed statistically significant positive correlations across all comparisons (r = 0.675 for V2, r = 0.656 for V3, r = 0.657 for V4; all *p* < 0.001), indicating that higher preoperative values were associated with higher postoperative outcomes. Linear regression models confirmed that V1 significantly predicted postoperative values, with R^2^ ranging from 0.43 to 0.46. The estimated statistical power of the study, assuming a medium effect size and α = 0.05, was 0.87, suggesting a high probability of detecting true effects. Higher preoperative breast stiffness was a predictor of increased postoperative stiffness. No significant differences were observed between postoperative days 7 and 14. No significant differences were noted between the left and right breasts at any time point (Mann–Whitney U test, *p* > 0.05).

No statistically significant correlations were found between breast stiffness (both pre- and postoperative) and Baker grade, nor between tonometric breast stiffness and elastographic tissue characteristics or maximum/minimum capsule thickness assessed at ≥1 year postoperatively (Spearman’s Rs, *p* > 0.05 for all comparisons).

Similarly to the tonometric findings, the elastographic characteristics of the individual breast tissues at the designated measurement points also changed compared to the preoperative state (Table 2). All examined tissues showed a statistically significant increase in stiffness postoperatively on V2 and V3 compared to V1, with most tissues also remaining stiffer at the 1-year follow-up (adipose and glandular tissue in the lower, inner and outer quadrants, as well as fascia and muscle in the upper quadrants). The remaining parameters showed no statistically significant differences from the preoperative state. No significant differences in tissue stiffness were observed between days 7 and 14, except for higher values in adipose tissue in the inner quadrant and lower values at the peri-implant region in the outer quadrant (Wilcoxon test, *p* < 0.05 for each comparison).

At the ≥12-month follow-up, elastography values for all tissue types and locations were significantly lower than those on days 7 and 14 (Wilcoxon test, *p* < 0.05 for each comparison). No significant differences in the analyzed parameters were found between the left and right breasts at any time point (V2–V4; Mann–Whitney U test, *p* > 0.05 in all cases).

No significant correlation was found between the baseline elastographic characteristics of the breast assessed preoperatively (V1) and the clinical Baker scale grading of the capsule (Spearman’s Rs, *p* > 0.05 in all cases). However, several positive and negative correlations were identified between the Baker scores at V4 and selected elastographic parameters measured on postoperative day 7 (V2), day 14 (V3) and at ≥1 year postoperatively (V4) (Table 3).

## 4. Discussion

The Baker classification remains the most widely used method for assessing complications related to silicone breast implants, especially capsular contracture (CC). Its grades range from I (soft, natural breast) to IV (hard, painful and visibly deformed breast) and is valued for its simplicity and practicality in clinical settings. However, the system has clear limitations. It is based entirely on a physician’s examination and is therefore subjective, with variability between different observers and poor reproducibility in some cases [8,10,23,24,25,33].

Capsular contracture occurs when the fibrous capsule around the implant thickens and tightens, increasing tension on the implant shell. This can change the implant’s shape, making it more elongated and firm. In clinical follow-up, Baker grading provides a quick and easy way to decide when surgery may be needed. Yet, in research and when comparing outcomes between studies, more objective measures are necessary. In the Baker scale, the assessment focuses only on the implant itself, evaluating characteristics such as firmness and pain on palpation, rather than the breast as a whole, which is what tonometry measures. In contrast, elastography provides an image of the breast-implant complex, taking into account its individual components such as the surrounding tissues separately, the implant and the interface between them.

Formation of capsular contracture as a complication after inserting the implants remains much more frequent (occurrence of over 10%) than spontaneous breakage or silicone gel leak [7,10]. Imaging techniques like ultrasound (US) and magnetic resonance imaging (MRI) have been proposed as useful tools to assess and predict capsular contracture. For the early detection of potential implant failure, in Japan, ultrasound and MRI are recommended every 2 years following breast reconstruction [7,34]. Studies suggest they correlate well across Baker grades I to IV and can add more consistency to the evaluation [10,25,33,35]. Imaging is particularly helpful in patients where physical findings are unclear or subtle. Torres-Uniga et al. showed that capsular thickness measured by ultrasound correlates with the clinical Baker scale, with thicker capsules in breasts graded III/IV, that five times more often had shell wrinkles on ultrasound compared to Baker I/II [odds ratio (OR), 5.25], although with *p* = 0.0496 [35]. Bui et al. showed significant difference in capsule thickness only between Baker I and II, III or IV, whereas the thickness of capsules in Baker II-IV did not differ significantly [9]. Although false-positive imaging diagnosis of implant failure are also reported [34].

Our results suggest that preoperative elastography cannot be used to estimate the risk of capsular contracture. However, early postoperative measurements (at 7 and 14 days and after one year) in certain quadrants and tissue regions showed some associations with Baker scores at one year, implying that tissue changes in the early healing period may be linked to later contracture, whereas tonometry demonstrated no correlation with the Baker scale in any region at any time.

To improve assessment, some researchers recommend combining clinical, imaging and also quantitative histology (histomorphometry) evaluation [8,9,25]. According to Pagani et al. [8] after clinically assessed breast deformation, ultrasonography revealed intracapsular rupture and thick fibrotic capsule, whereas MRI showed bulge of the prosthesis without a rupture. After a core needle biopsy and chest radiography, fibrous calcium deposits and capsular calcification were found [8]. A reliable classification using the Baker score was not possible, as the patient was asymptomatic. Histology demonstrates a transformation of capsule fibers from thin and loosely packed to dense, reflecting the process of capsule thickening [25], although invasive nature limits its routine clinical use.

Monitoring changes over time can also help capture how CC progresses and reduce errors from single time point assessments. Baker grading remains the most common approach, mainly because it is simple and low-cost. However, it is sometimes applied in situations where true capsular formation may not occur—for example, in prepectoral reconstructions using acellular dermal matrices (ADM). In these cases, any “contracture” may actually result from fibrosis of the skin, fat, ADM or muscle, rather than the capsule itself [25]. Ruffenach et al. [36] observed that the overall stiffness of the implant is significantly higher after 8 years of implantation. An implant implanted for 17.5 years is three times stiffer than one implanted for just over 3 years [36].

Overall, while Baker grading is still a valuable tool in daily practice, its limitations highlight the need for more standardized and objective methods for evaluating capsular contracture, especially in complex reconstructions and research settings. Although tonometry did not correlate with Baker grade, we observed a general increase in stiffness values postoperatively. This may be attributed to transient postoperative factors such as edema, implant size, and individual breast volume, which can influence surface tension without necessarily indicating capsular contracture severity. Moreover, applanation tonometry may lack the sensitivity to detect subtle internal tissue changes, as it primarily measures surface compliance. Previous studies have emphasized that tonometry alone may not be sufficient for clinical assessment and should be complemented by other objective or clinical grading methods [25,37]. Still, no single method has yet proven to be a fully reliable and specific way to measure the degree of contracture. This study represents an attempt to expand diagnostic methods for assessing and predicting the development of capsular contracture.

2D shear wave elastography (SWE) is limited by lesion heterogeneity and its two-dimensional view, while 3D SWE provides automatic volumetric assessment. Combining 2D + 3D SWE with conventional breast ultrasound may reduce unnecessary biopsies in BI-RADS 4–5 lesions [28,29,30]. While not specifically focused on capsular formation, elastography has shown value as an adjunct tool for improving diagnostic accuracy in breast evaluations, as confirmed in multiple studies [22,26,27,28,30,38,39]. On animal model, strain elastography provided more reliable data than SWE for differentiating between intact and ruptured implants [40]. In our study 2D SWE with good reproducibility was enough and successful to prove differences from the preoperative state for selected parameters. The correlations observed between SWE measurements and Baker scores are weak and should be interpreted as associations rather than validated predictive relationships.

However, in a prospective study of 11 patients with breast implants, capsular contracture severity was assessed clinically using the Baker score and correlated with ultrasound elastography measurements [33]. The findings demonstrated a strong correlation between the Baker grade and elastography results (κ = 0.83–0.89). The discrepancies in findings could be attributed to the greater heterogeneity of the cohort in the study by Prantl or to the relatively small sample size. Capsular tissue in patients with grades 3 and 4 contracture in Baker’s classification was significantly thicker compared to those with grade 2 contracture [41]. Their another study demonstrated that patients with capsular contracture had markedly elevated serum hyaluronan levels compared to controls (26 ± 14 μg/L vs. 12 ± 6 μg/L; *p* < 0.05). Moreover, a strong positive relationship was identified between the severity of capsular contracture (Baker 1–4) and serum hyaluronan concentrations (grades 1–2: 15 ± 3 μg/L; grades 3–4: 35 ± 12 μg/L; r^2^ = 0.73; *p* < 0.05) [41].

The increased stiffness detected by shear wave elastography and tonometry in the early postoperative period likely reflects fibrotic remodeling driven by cellular and molecular mechanisms. Capsular contracture is associated with the activation of myofibroblasts, characterized by α-smooth muscle actin (α-SMA) expression, which contract the extracellular matrix and increase tissue rigidity. Central to this process is TGF-β1 signaling, which stimulates myofibroblast differentiation and promotes collagen synthesis and deposition [7,8,42,43,44,45].

Thus, regions showing higher stiffness in our measurements may correspond to areas with enhanced TGF-β1-mediated fibrotic activity, increased α-SMA-positive myofibroblast infiltration and denser collagen networks. Recent consensus reports on BIA-ALCL management emphasize the pivotal role of chronic periprosthetic inflammation in lymphomagenesis and capsular pathology, supporting the concept that persistent immune activation around implants can lead to fibrotic and neoplastic transformation [46]. By quantifying mechanical properties noninvasively, elastography and tonometry may capture these early fibrotic changes before they become clinically evident, providing an objective surrogate of the underlying molecular and cellular processes that ultimately drive capsular contracture.

The etiology of capsular contracture is multifactorial, with one of the widely recognized causes being biofilm formation due to contamination of the implant or pocket by skin flora [15,16,17]. Smooth-surface implants in theory allow for less bacterial adhesion compared to textured implants, which could potentially reduce the risk of capsular contracture. However, meta-analyses have shown that textured breast implants are actually associated with lower rates of this complication [6,7,12]. The source of bacteria or biofilm on the implant surface may include the skin or nipple, as the milk ducts are believed to be colonized by various bacteria [17]. The study of Schreml et al. [15] examined 45 women with unilateral capsular contracture after breast augmentation to investigate the role of bacterial colonization. Bacteria were detected in 66.7% of high-grade contractures (Baker III/IV) but not in low-grade cases (Baker I/II), showing a significant difference between the groups (*p* < 0.001) [15]. These findings suggest that bacterial contamination may contribute to inflammation and fibrosis in severe capsular contracture. It has been proved that using a Keller funnel, which minimizes implant contact with the skin and potential contamination, significantly reduces the rate of reoperations for capsular contracture within 12 months after primary breast augmentation [16,17,47].

Another approach to reducing capsular contracture was suggested by Sang et al., noting that the cellular composition of mature capsules resembles that of scar tissue, which, as is widely known, can be treated with botulinum neurotoxin type A (BoNT-A) [48]. BoNT-A inhibits TGF-ß1 signaling, preventing fibroblast differentiation into myofibroblasts and thereby reducing capsule formation. In animal studies, this effect was shown to be dose-dependent. The suggested timing for US and MRI screening in asymptomatic patients with implants is 5–6 years after surgery, followed by repeat imaging every 2–3 years [49,50]. Bogdan et al. emphasized the need for standardized imaging protocols that account for physiological variables such as respiratory state, menstrual cycle and patient posture, as these factors can influence breast volume measurements [22]. In contrast, another study assessing breast tissue elasticity throughout the menstrual cycle found no statistically significant changes in mean glandular or fat tissue elasticity, suggesting that while breast volume may fluctuate, its elastic properties remain relatively stable [31].

### Limitations

This study has several limitations that should be acknowledged. The **small sample size (*n* = 28)** inevitably limits the statistical power to detect strong correlations, especially when the studied phenomena (capsular stiffness and clinical contracture) evolve over time and are influenced by multiple factors. **The timing of the measurements** (postoperative days 7, 14, and over 12 months) may not fully capture the dynamic process of capsule maturation. Subtle stiffness changes occurring between these points could have been missed. The **Baker score itself is a subjective clinical grading system**, which may introduce variability not directly related to tissue mechanical properties detected by SWE or tonometry. Future studies incorporating biopsy or histomorphometric analysis could provide deeper insight into the biological mechanisms driving capsular contracture.

## 5. Conclusions

Breast stiffness, as measured by tonometry and shear wave elastography, can be successfully used for monitoring during follow-up after breast implant surgery, rather than as predictive tools. Unlike tonometry, sonoelastography allows precise evaluation of stiffness across all tissue layers. SWE partially correlated with the modified Baker scale for capsular contracture. Although our results suggest that preoperative SWE cannot predict capsular contracture, early postoperative changes—particularly those observed at 7 and 14 days—may hold clinical relevance. If specific elastographic patterns are consistently associated with higher Baker scores at one year, this could justify closer monitoring of patients showing such changes. For example, patients with increased stiffness in certain quadrants might benefit from more frequent imaging follow-up or early therapeutic interventions, such as anti-inflammatory treatment or timely physiotherapy, aimed at modulating tissue remodeling. Incorporating SWE into early postoperative protocols could thus support a more personalized approach to implant surveillance and potentially reduce the incidence or severity of contracture.

## Figures and Tables

**Table 1:** Tonometric assessment (N/cm^2^) in the study group.

	Mean ± SD
**Before surgery (V1)**	625.0 ± 159.6
**On postoperative day 7 (V2)**	1021.0 ± 238.2
**On postoperative day 14 (V3)**	979.0 ± 244.2
**>1 year after surgery (V4)**	765.6 ± 142.8

**Table 2:** Elastographic characteristics (kPa) of the study group.

		V1 Preoperative	V2 Day 7 Postoperative	V3 Day 14 Postoperative	V4 >1 Year Postoperative
		Mean ± SD			
**Adipose Tissue**	**LQ**	4.7 ± 2.3	12.0 ± 7.6 *	11.7 ± 6.2 *	7.7 ± 4.6 *
	**UQ**	7.8 ± 4.3	11.9 ± 4.7 *	10.5 ± 5.1 *	7.6 ± 4.2
	**OQ**	6.7 ± 3.8	14.6 ± 6.3 *	13.4 ± 6.5 *	10.5 ± 5.7 *
	**IQ**	7.7 ± 5.1	12.9 ± 5.4 *	15.0 ± 7.7 *	9.1 ± 4.0 *
**Fascia**	**UQ**	9.2 ± 4.5	13.3 ± 4.9 *	13.2 ± 5.8 *	11.2 ± 4.7 *
	**IQ**	12.0 ± 7.3	22.8 ± 11.8 *	20.5 ± 6.3 *	13.2 ± 4.4
**Muscle**	**UQ**	9.4 ± 5.2	14.5 ± 5.8 *	15.9 ± 7.3 *	11.4 ± 5.7 *
	**IQ**	10.6 ± 5.5	22.8 ± 11.8 *	21.8 ± 7.4 *	14.4 ± 4.8
**Glandular Tissue**	**LQ**	5.8 ± 3.0	14.3 ± 6.9 *	12.1 ± 7.9 *	9.0 ± 4.3 *
	**UQ**	8.7 ± 3.7	12.9 ± 4.7 *	12.6 ± 6.0 *	8.5 ± 3.7
	**OQ**	9.5 ± 12.9	15.0 ± 6.7 *	14.4 ± 5.9 *	11.7 ± 5.9 *
	**IQ**	8.4 ± 4.2	16.1 ± 6.9 *	16.5 ± 6.6 *	11.3 ± 4.4 *
**Peri-implant region (capsule)**	**LQ**	-	23.7 ± 7.7	22.4 ± 6.7	15.1 ± 4.8
	**UQ**	-	19.6 ± 5.5	18.3 ± 6.1	13.4 ± 5.0
	**OQ**	-	25.5 ± 8.9	22.5 ± 7.5	17.7 ± 6.4
	**IQ**	-	25.4 ± 8.7	23.5 ± 6.6	15.7 ± 4.9

* statistically significant difference compared to the preoperative level (*p* < 0.05, Wilcoxon test). LQ—lower quadrants, UQ—upper quadrants, OQ—outer quadrants, IQ—inner quadrants.

**Table 3:** Correlations (Spearman’s Rs, *p*-values, 95% CI) between elastographic parameters assessed at ≥1 year postoperatively and the clinical evaluation of the capsule according to the Baker scale.

		Baker Scale		
		Late Postoperative Elastography to Endpoint Baker Scale (V4)	Early Postoperative Elastography to Endpoint Baker Scale	
		V4	V2	V3
**Adipose** **Tissue**	**LQ**	Rs = 0.01 *p* = 0.883 CI = (−0.361, 0.413)	Rs = 0.093 *p* = 0.652 CI = (−0.306, 0.463)	Rs = 0.348 *p* = 0.082 CI = (−0.046, 0.710)
	**UQ**	Rs = 0.06 *p* = 0.846 CI = (−0.421, 0.353)	Rs = −0.105 *p* = 0.609 CI = (−0.473, 0.294)	Rs = 0.241 *p* = 0.236 CI = (−0.158, 0.618)
	**OQ**	Rs = 0.02 *p* = 0.730 CI = (−0.325, 0.446)	Rs = −0.040 *p* = 0.845 CI = (−0.421, 0.352)	Rs = 0.376 *p* = 0.058 CI = (0.001, 0.665)
	**IQ**	**Rs = 0.46** ***p* = 0.014** **CI = (0.109, 0.729)**	Rs = −0.145 *p* = 0.481 CI = (−0.504, 0.257)	Rs = 0.235 *p* = 0.249 CI = (−0.197, 0.602)
**Fascia**	**UQ**	Rs = 0.10 *p* = 0.512 CI = (−0.267, 0.496)	Rs = 0.377 *p* = 0.058 CI = (−0.012, 0.667)	Rs = 0.199 *p* = 0.329 CI = (−0.199, 0.544)
	**IQ**	**Rs = 0.28** ***p* = 0.042** **CI = (−0.018, 0.663)**	Rs = −0.138 *p* = 0.501 CI = (−0.499, 0.263)	Rs = 0.316 *p* = 0.116 CI = (−0.102, 0.678)
**Muscle**	**UQ**	Rs = −0.01 *p* = 0.938 CI = (−0.401, 0.374)	**Rs = 0.388** ***p* = 0.050** **CI = (0.001, 0.674)**	Rs = 0.070 *p* = 0.734 CI = (−0.348, 0.454)
	**IQ**	Rs = 0.25 *p* = 0.184 CI = (−0.132, 0.595)	Rs = −0.131 *p* = 0.524 CI = (−0.493, 0.270)	Rs = 0.248 *p* = 0.221 CI = (−0.178, 0.655)
**Glandular** **Tissue**	**LQ**	Rs = 0.12 *p* = 0.612 CI = (−0.295, 0.473)	Rs = 0.305 *p* = 0.130 CI = (−0.093, 0.619)	Rs = 0.321 *p* = 0.110 CI = (−0.181, 0.716)
	**UQ**	Rs = 0.08 *p* = 0.964 CI = (−0.395, 0.379)	Rs = 0.168 *p* = 0.411 CI = (−0.234, 0.522)	Rs = 0.182 *p* = 0.374 CI = (−0.259, 0.572)
	**OQ**	Rs = 0.05 *p* = 0.892 CI = (−0.363, 0.411)	Rs = 0.008 *p* = 0.970 CI = (−0.381, 0.394)	**Rs = 0.436** ***p* = 0.026** **CI = (0.088, 0.686)**
	**IQ**	Rs = 0.18 *p* = 0.134 CI = (−0.097, 0.617)	Rs = −0.136 *p* = 0.508 CI = (−0.497, 0.265)	Rs = 0.303 *p* = 0.132 CI = (−0.178, 0.710)
**Peri-implant region**	**LQ**	Rs = 0.08 *p* = 0.754 CI = (−0.331, 0.441)	Rs = 0.063 *p* = 0.759 CI = (−0.332, 0.440)	Rs = 0.121 *p* = 0.557 CI = (−0.276, 0.492)
	**UQ**	Rs = −0.06 *p* = 0.370 CI = (−0.533, 0.220)	Rs = 0.089 *p* = 0.665 CI = (−0.309, 0.461)	Rs = 0.016 *p* = 0.939 CI = (−0.372, 0.386)
	**OQ**	Rs = −0.08 *p* = 0.370 CI = (−0.644, 0.520)	Rs = −0.211 *p* = 0.301 CI = (−0.553, 0.192)	Rs = 0.218 *p* = 0.284 CI = (−0.271, 0.637)
	**IQ**	**Rs = 0.33** ***p* = 0.047** **CI = (−0.013, 0.667)**	Rs = −0.101 *p* = 0.625 CI = (−0.470, 0.298)	Rs = 0.308 *p* = 0.125 CI = (−0.160, 0.693)

LQ—lower quadrants, UQ—upper quadrants, OQ—outer quadrants, IQ—inner quadrants.

## Data Availability

The data are available upon reasonable request.

## Abbreviations

The following abbreviations are used in this manuscript:

SWE	Shear-wave Elastography
SE	Strain Elastography
CC	Capsular contracture
FDA	Food and Drug Administration
